# Mixed Invasive Fungal Pneumonia Caused by *Aspergillus*, *Candida*, *Mucor* in an Immunocompromised Host

**DOI:** 10.1002/rcr2.70775

**Published:** 2026-10-29

**Authors:** Taeyun Kim, Eunjoo Jung, Yohan Jeon, Hyunji Choi, Ho‐Sup Lee, Tae‐Won Jang, Chul‐Ho Oak

**Affiliations:** ^1^ Division of Pulmonology, Department of Internal Medicine Kosin University Gospel Hospital, Kosin University College of Medicine Busan Republic of Korea; ^2^ Department of Internal Medicine Kosin University Gospel Hospital Busan Republic of Korea; ^3^ Department of Pathology Kosin University Gospel Hospital, Kosin University College of Medicine Busan Republic of Korea; ^4^ Department of Laboratory Medicine Kosin University Gospel Hospital, Kosin University College of Medicine Busan Republic of Korea; ^5^ Division of Hematology & Oncology, Department of Internal Medicine Kosin University Gospel Hospital, Kosin University College of Medicine Busan Republic of Korea

**Keywords:** *Aspergillus*, *Candida*, immunocompromised, mucormycosis, pneumonia

## Abstract

Invasive fungal pneumonia (IFP) is a life‐threatening complication in immunocompromised patients, particularly those with hematologic malignancies. Mixed IFP are uncommon, given its substantial diagnostic and therapeutic challenges. We report a case of rapidly progressive mixed IFP caused by *Aspergillus*, *Candida* and *Mucor* species in a 60‐year‐old man with diffuse large B‐cell lymphoma following chemotherapy. Despite early empirical broad‐spectrum antibiotics and prompt initiation of antifungal therapy guided by serum galactomannan positivity and radiologic progression, the patient's clinical condition deteriorated. Bronchoscopy revealed extensive necrotic endobronchial lesions, and histopathologic examination confirmed simultaneous infection with three fungal pathogens. Although intensive treatment with an antifungal agent and mechanical ventilation were provided, the patient died of refractory respiratory failure within 1 week of hospitalization. This case highlights that clinicians should actively consider mixed fungal infection as a potential aetiology of IFP in immunocompromised patients, rather than assuming a single‐pathogen process.

## Graphical abstract

We report a case of mixed invasive fungal pneumonia caused by *Aspergillus*, *Candida* and *Mucor* species in an immunocompromised host, highlighting the diagnostic and therapeutic implications of concurrent opportunistic fungal infections.

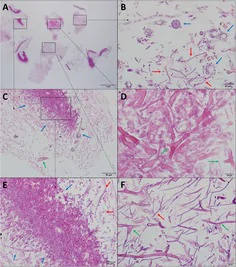

## 1 Introduction

Invasive fungal pneumonia (IFP) has become increasingly common in vulnerable populations, including patients receiving chemotherapy, those with acquired immunodeficiency syndrome, neoplastic disease, advanced age and individuals on immunosuppressive therapy [1, 2]. In these settings, simultaneous infection by more than one fungal pathogen may be mistaken for a single‐organism infection, which can delay appropriate treatment and adversely affect clinical outcomes. Patients with IFP caused by multiple fungal species experience high morbidity and mortality, reflecting differences in antifungal susceptibility among pathogens [3]. However, accurately identifying multiple fungal aetiologies remains challenging, as conventional histopathology has limited discriminatory power and the application of molecular diagnostic methods to formalin‐fixed tissue is technically demanding [4]. In this context, we report a case of mixed IFP caused by *Aspergillus*, *Candida* and *Mucor* species in an immunocompromised host, highlighting the diagnostic and therapeutic implications of concurrent opportunistic fungal infections.

## 2 Case Report

A 60‐year‐old male with a recent diagnosis of diffuse large B‐cell lymphoma (DLBCL) presented to the emergency department with a drowsy mental state, 5 days after receiving his second cycle of rituximab, cyclophosphamide, doxorubicin, vincristine and prednisone (R‐CHOP) therapy. Upon arrival, the patient was intubated for airway protection due to his deteriorating neurological status.

Initial laboratory findings revealed neutrophilia (10,910 cells/μL), elevated C‐reactive protein (18.255 mg/dL) and interleukin‐6 (461.53 pg/mL), all suggesting an ongoing inflammatory response, likely due to infection. A chest computed tomography (CT) scan revealed pneumonic consolidation in the right upper and lower lobes (RUL and RLL) (Figure 1A,B). However, there were no radiologic signs suggestive of fungal pneumonia, such as halo signs or cavitary lesions, making the diagnosis of fungal infection less likely at first glance.

**FIGURE 1:**
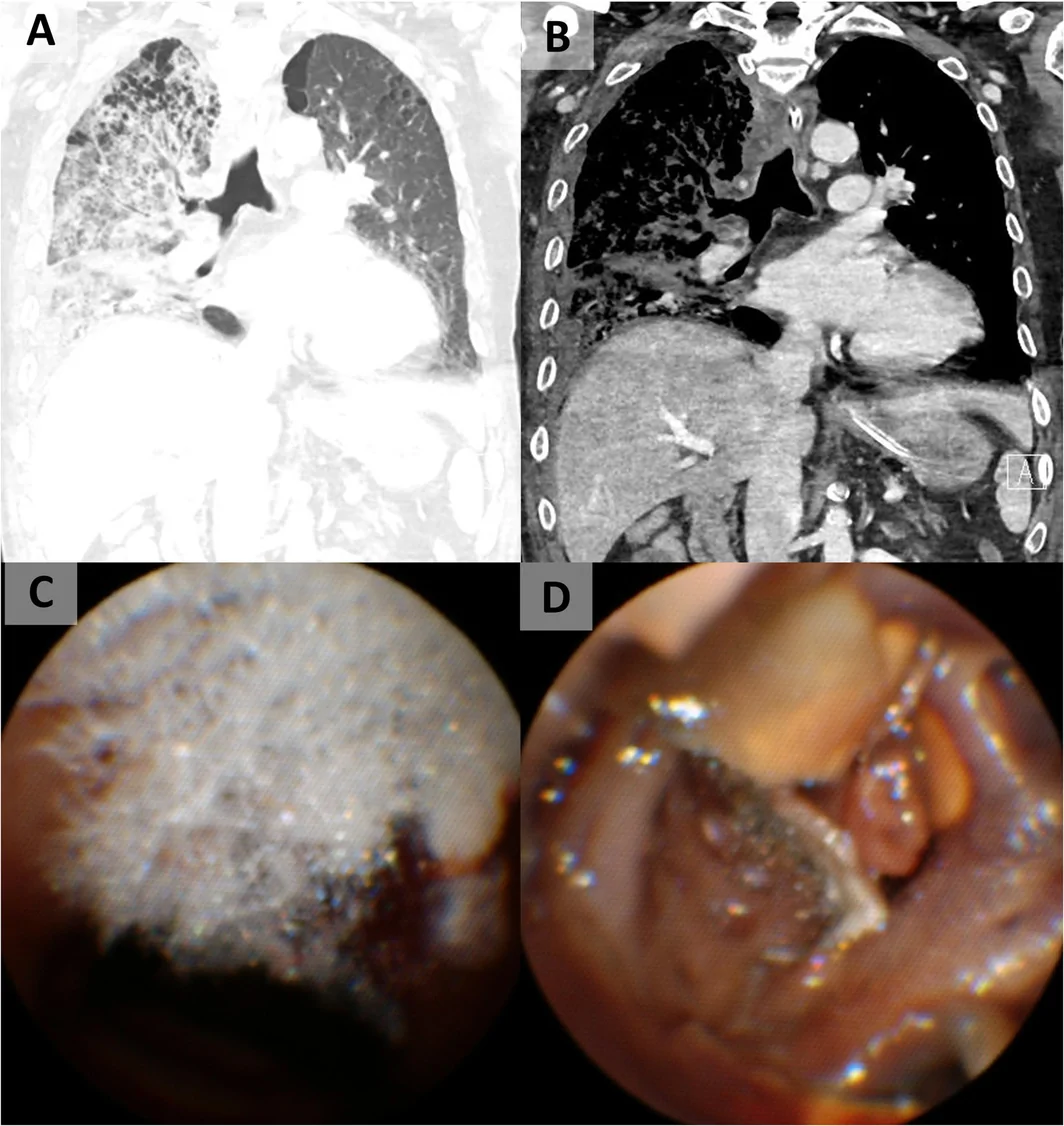
Radiologic and bronchoscopic findings. (A, B) Chest computed tomography (CT) with lung window setting shows extensive consolidation with surrounding ground‐glass opacities and irregular infiltrative lesions predominantly involving the right lung, suggestive of extensive pneumonia. (C) Bronchoscopic examination of the right upper lobe (RUL) shows diffuse whitish plaques with friable mucosal surfaces and adherent debris, (D) closer bronchoscopic view of the RUL reveals extensive mucosal necrosis and pseudomembrane‐like lesions obstructing the bronchial lumen.

Empirical broad‐spectrum antibiotic therapy was started immediately, including meropenem, levofloxacin and teicoplanin, to cover a wide range of bacterial pathogens. Granulocyte‐colony stimulating factor (G‐CSF) was administered to manage the severe pancytopenia noted on subsequent laboratory tests, likely secondary to the chemotherapy regimen. Despite aggressive antimicrobial treatment, the patient remained febrile with persistently elevated C‐reactive protein levels. Serial chest radiographs showed progressive worsening of pneumonia, raising further suspicion of a non‐bacterial aetiology.

On hospital Day 4, serum galactomannan testing was found to be positive, and sputum cultures grew mould‐like fungi, prompting the initiation of voriconazole for suspected invasive aspergillosis.

Given the patient's worsening condition and partial recovery from his pancytopenia, bronchoscopy was performed. The procedure revealed diffuse whitish plaques with friable mucosal surfaces at the RUL bronchial orifice (Figure 1C). Upon closer inspection, extensive mucosal necrosis and pseudo‐membrane‐like lesions obstructing the bronchial lumen were noted (Figure 1D), which are highly suggestive of invasive fungal infection. Bronchoalveolar lavage culture grew *Aspergillus fumigatus*. Histopathologic examination of the endobronchial biopsy revealed fungal elements morphologically consistent with *Aspergillus*, *Candida* and *Mucor* (Figure 2A–F). Species‐level identification of *Candida* and *Mucor* was not available. The histopathologic findings indicating concomitant infection were reported 1 day before the patient's death. These findings established the diagnosis of mixed IFP.

**FIGURE 2:**
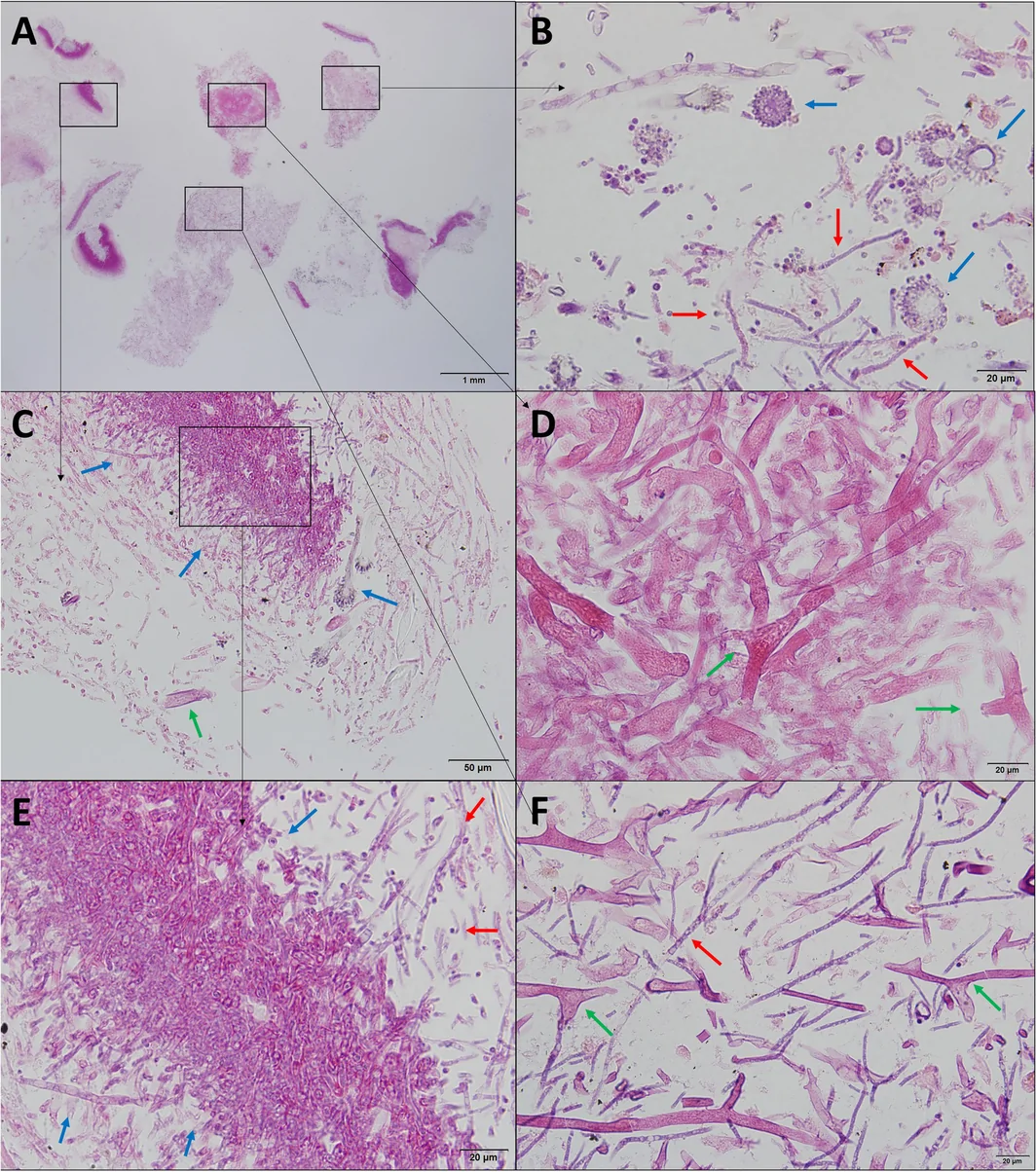
Microscopic findings. (A) scan view of the biopsy sample, (B) mixed *Aspergillus* (blue arrow) and *Candida* (red arrow), (C) mixed *Aspergillus* (blue arrow) with zonal phenomenon and mucormycetes (green arrow), (D) mucormycetes showing non‐septate, irregularly broad hyphae with right‐angle branching, (E) mixed *Aspergillus* (blue arrow) and *Candida* (red arrow), (F) mixed mucormycetes (green arrow) and *Candida* (red arrow).

Despite prompt initiation of voriconazole and supportive care, including intubation and mechanical ventilation for respiratory support, the patient's condition deteriorated rapidly. His pneumonia progressed, and he died of respiratory failure on hospital Day 8.

## 3 Discussion

IFP remains a life‐threatening condition, particularly in immunocompromised patients, and early identification of causative pathogens and initiation of appropriate antifungal therapy continue to pose significant clinical challenges. Despite advances in diagnostic modalities and antifungal agents, mortality associated with IFP remains high, especially among patients with immune suppression [2]. While most cases of IFP are approached under the assumption of a single fungal pathogen, the possibility of concurrent infection with multiple fungal species should not be overlooked, as such co‐infections may be associated with rapid clinical deterioration and poor outcomes.

Diagnosing mixed fungal infection is inherently challenging due to the substantial overlap in clinical presentation and radiologic features among different fungal pneumonias. Findings such as nodules, consolidations, cavitary lesions or halo signs are not pathogen‐specific and may be indistinguishable across opportunistic infections caused by fungi [4]. Moreover, current diagnostic strategies often rely on the identification of a dominant organism through culture, histopathology or fungal biomarkers, which may inadvertently bias clinicians toward a single‐pathogen diagnosis. Empirical antifungal therapy initiated after isolation of one fungal species may therefore lead to premature diagnostic closure, delaying recognition of co‐existing fungi with distinct antifungal susceptibility profiles.

From a microbiological and pathophysiological perspective, mixed fungal infections are associated with an aggressive clinical course that likely reflects synergistic mechanisms of tissue invasion, immune evasion and treatment failure [3]. In addition, antifungal regimens effective against one organism may provide inadequate coverage against another, particularly in combinations involving *Mucorales* or intrinsically resistant *Candida* species. In immunocompromised hosts, this mismatch between pathogen burden and antifungal coverage can result in rapid respiratory deterioration and high mortality, even when antifungal therapy is initiated promptly and appropriately based on initial microbiologic data.

Importantly, emerging literature suggests that mixed fungal infections may be underreported rather than truly rare, especially in patients with profound or prolonged immunosuppression. Advances in molecular diagnostics, including multiplex PCR and next‐generation sequencing, have begun to reveal a higher‐than‐expected prevalence of polymicrobial fungal infections in invasive disease [1, 4]. These findings support the notion that conventional culture‐based methods alone may underestimate the true complexity of fungal pneumonia in high‐risk populations.

In conclusion, this case emphasizes the importance of maintaining a high index of suspicion for mixed fungal IFP, particularly in immunocompromised patients. Comprehensive and timely diagnostic evaluation—including repeated sampling, histopathologic confirmation and consideration of molecular techniques—should be pursued. Recognizing the possibility of concurrent fungal pathogens is essential to avoid delayed diagnosis, guide appropriate antifungal selection and ultimately improve clinical outcomes in this highly vulnerable population.

## Author Contributions

**Taeyun Kim:** writing – original draft, data curation, validation. **Eunjoo Jung:** writing – original draft, validation. **Yohan Jeon:** validation. **Hyunji Choi:** validation. **Ho‐Sup Lee:** validation. **Tae‐Won Jang:** supervision, project administration, writing – review and editing. **Chul‐Ho Oak:** supervision, validation.

## Funding

The authors have nothing to report.

## Ethics Statement

This case report was conducted in accordance with the Declaration of Helsinki. According to the policies of the Institutional Review Board of Kosin University Gospel Hospital, ethical approval was waived because this manuscript describes a retrospective case involving a deceased patient, and all clinical data and images were fully anonymized.

## Consent

The authors declare that written informed consent was obtained for the publication of this manuscript and accompanying images from the patient's next of kin using the form provided by the journal.

## Conflicts of Interest

The authors declare no conflicts of interest.

## Data Availability

The data that support the findings of this study are available on request from the corresponding author. The data are not publicly available due to privacy or ethical restrictions.
